# Ichthyofauna in the last free-flowing river of the Lower Iguaçu basin: the importance of tributaries for conservation of endemic species

**DOI:** 10.3897/zookeys.1041.63884

**Published:** 2021-06-03

**Authors:** Suelen Fernanda Ranucci Pini, Maristela Cavicchioli Makrakis, Mayara Pereira Neves, Sergio Makrakis, Oscar Akio Shibatta, Elaine Antoniassi Luiz Kashiwaqui

**Affiliations:** 1 Instituto Federal de Mato Grosso do Sul (IFMS), Rua Salime Tanure s/n, Santa Tereza, 79.400-000 Coxim, MS, Brazil Instituto Federal de Mato Grosso do Sul Coxim Brazil; 2 Grupo de Pesquisa em Tecnologia em Ecohidráulica e Conservação de Recursos Pesqueiros e Hídricos (GETECH), Programa de Pós-graduação em Engenharia de Pesca, Universidade Estadual do Oeste do Paraná (UNIOESTE), Rua da Faculdade, 645, Jardim La Salle, 85903-000 Toledo, PR, Brazil Universidade Estadual do Oeste do Paraná Toledo Brazil; 3 Programa de Pós-Graduação em Biologia Animal, Laboratório de Ictiologia, Departamento de Zoologia, Instituto de Biociências, Universidade Federal do Rio Grande do Sul (UFRGS), Avenida Bento Gonçalves, 9500, Agronomia, 90650-001, Porto Alegre, RS, Brazil Universidade Federal do Rio Grande do Sul Porto Alegre Brazil; 4 Departamento de Biologia Animal e Vegetal, Universidade Estadual de Londrina, Rod. Celso Garcia Cid PR 445 km 380, 86057-970, Londrina, PR, Brazil Universidade Estadual de Londrina Londrina Brazil; 5 Grupo de Estudos em Ciências Ambientais e Educação (GEAMBE), Universidade Estadual de Mato Grosso do Sul (UEMS), Br 163, KM 20.7, 79980-000 Mundo Novo, MS, Brazil Universidade Estadual de Mato Grosso do Sul Mundo Novo Brazil

**Keywords:** Abundance, fish, origin, richness, size, threats, updated list

## Abstract

The fish fauna from the Lower Iguaçu River and tributaries upstream of the Iguaçu Falls, the last free-flowing river stretch, were investigated. Twenty five sites in tributaries and the main channel were sampled between 2010 and 2016 using several kinds of fishing gear. The species were categorized according to their size, origin, and conservation status. Species richness and abundance in the main channel and tributaries were compared. In total, 87,702 specimens were recorded, comprising 76 species, 25 families, 53 genera, and eight orders. Characiformes and Siluriformes were the richest orders, representing 92% of the total specimens; Characidae, Cichlidae, Pimelodidae, and Loricariidae were the richest families. The fish fauna was composed of small and medium-sized species and included endemic (42%), autochthonous (24%), allochthonous (21%), and exotic (9%) species, as well as hybrids (4%). Significant differences in the relative numerical abundance of species were found among sites. *Ancistrus mullerae* and *Rhamdia branneri* (endemic) were indicator species for tributaries inside of Iguaçu National Park (INP), while *Phalloceros harpagos* (autochthonous) and *Ictalurus punctatus* (exotic) for tributaries outside of INP and *Odontesthes bonariensis* (allochthonous) for the main channel. The last dam-free stretch of the Lower Iguaçu River and tributaries upstream the Iguaçu Falls exhibits a rich endemic fish fauna, including some rare, endangered species (*Steindachneridion melanodermatum*, *Gymnogeophagus taroba*, and *Psalidodon gymnogenys*). These findings are essential to predict and understand the effects caused by the new Baixo Iguaçu Hydroelectric Power Plant and highlight the importance of tributaries and Iguaçu National Park for conservation of endemic species.

## Introduction

The high diversity of species in the Neotropical region is recognized worldwide. This region currently has more than 5,160 species of freshwater fish and may have as many as 9,000 species (Reis et al. 2016). Three large freshwater basins dominate the South American continent: Amazon, Orinoco, and Paraná-Paraguay (Reis et al. 2016). The Paraná-Paraguay basin represents the third most diverse freshwater basin in South America (Reis et al. 2016), and within it, the Iguaçu River is renowned for its peculiar geomorphological and ichthyofaunal characteristics (Baumgartner et al. 2012).

Endemism is a well-recognized feature of the Iguaçu river basin (Baumgartner et al. 2012), which has led to its classification as a distinct ecoregion for freshwater fish conservation (Abell et al. 2008). This unique fauna arose from the isolation of this basin caused by the formation of the Iguaçu Falls some 22 million years ago (Oligo-Miocene period) (Severi and Cordeiro 1994). Currently, approximately 127 species of fish are known from the Iguaçu river basin (Reis et al. 2020). Many of these species have been described in the last decade, although taxonomic problems remain (Baumgartner et al. 2012), indicating that the diversity may be underestimated.

The main anthropogenic threats to fish fauna are habitat loss and environmental degradation. Specifically, damming rivers for hydroelectric power generation and water diversion for irrigation, as well as extensive changes in land use for agriculture and urbanization, are the main drivers of habitat loss (Reis et al. 2016) and the leading causes of the loss of biodiversity (Carvalho et al. 2019; Teresa and Casatti 2017). Therefore, it is essential to identify species, understand their distribution, and mitigate threats.

The topographic relief of the Iguaçu river basin has been a major attraction for hydroelectric projects. There are now five large reservoirs and several small ones, which have changed the natural landscape and stream habitats in the basin (Baumgartner et al. 2012). The last dam-free stretch of the Iguaçu River is 190 km in length and extends downstream from Salto Caxias dam to the Iguaçu Falls and encompasses Iguaçu National Park (INP), a world heritage site. However, in 2013, construction began on the sixth hydroelectric power plant, the Baixo Iguaçu Hydroelectric Power Plant (HPP) about 30 km downstream of the Salto Caxias dam and 500 m upstream from the mouth of the Gonçalves Dias River, which forms the boundary of INP. INP is one of the few remaining areas of Atlantic Forest protected by law. Although this hydroelectric plant project is very controversial due to its possible impacts on the region and particularly on INP, its operation started in 2019. Worryingly, the Baixo Iguaçu HPP potentially could be source of threats to the fish fauna, especially endemic species both inside and outside the INP (UNESCO 2012; Assumpção et al. 2017; Delariva et al. 2018).

The demand for electricity has grown in recent decades. To supply this demand in Brazil, most of needed electricity comes from hydroelectric plants (Kliemann and Delariva 2015; Makrakis et al. 2019). The extensive water network favors the implementation of hydroelectric projects, from small and medium-sized plants to large ones, but these projects directly change the physical and abiotic characteristics of aquatic ecosystems (Barbosa et al. 1999; Pelicice et al. 2018) and their fauna. Among the adverse effects is the profound change in river hydrology, which alters the structure of the fish fauna by fragmenting habitat, restricting dispersal of fish, decreasing the diversity of microhabitats and the supply of resources, and preventing movements of migratory species (e.g., Agostinho et al. 2007). Effects on the trophic structure of fish are already known on the Iguaçu River at the Salto Caxias HPP (Delariva et al. 2013) and Salto Segredo HPP (Mise et al. 2013). Although these effects are recognized, cascade hydroelectric projects have become increasingly common in Brazilian rivers (Santos et al. 2018).

Changes in land use have also negatively affected the biodiversity of fish in the Lower Iguaçu river basin (Larentis et al. 2016; Delariva et al. 2018), and the Iguaçu River is also recognized as the second most polluted river in Brazil (Bueno-Krawczyk et al. 2015; IBGE 2015). This pollution originates mainly from industrial and domestic sewage of urban areas in the Higher Iguaçu region (Bueno-Krawczyk et al. 2015) and from contamination by pesticides using in agriculture in the middle and lower portions of the basin (Nimet et al. 2017; Neves et al. 2018). These threats can lead to species extinctions and changes in the distinct structure of the fish fauna, whose evolutionary and biogeographic history is still not well understood. Therefore, it is essential to study the fish fauna prior to additional anthropogenic threats to assess the state of this ecosystem’s conservation.

This study provides an ichthyofaunistic inventory of the last free-flowing river stretch of the Lower Iguaçu River. This area is poorly studied and may be affected by the construction of a new hydroelectric power plant near Iguaçu National Park. While a previous inventory has been carried out in the river mostly upstream of the Salto Caxias Dam (Baumgartner et al. 2012), our study was based on a wider spatial-temporal scale, and intense sampling efforts include areas not yet sampled downstream of this dam. The 190 km stretch of the Iguaçu River and its tributaries exhibits a diverse landscape, and includes the area protected within INP, including a pristine river, areas at the INP border, and anthropogenic areas. We compare the composition, richness, frequency, and numerical abundance of species in tributaries and the main river channel. We describe the relative numerical abundance of species according to their biogeographic origins among the sites. We determine fish species indicative for the main channel, as well as tributaries inside and outside of INP. The results contribute to the knowledge of the basin’s fish fauna, including important information on the biogeographic origins and conservation status of the species. Our new data are an important contribution to the conservation and sustainable management of the last free-flowing stretch of the Lower Iguaçu River and mitigate future anthropogenic threats to this river’s fish fauna.

## Material and methods

### Study area

The Iguaçu River is considered one of the most important tributaries of the Paraná river basin, having 1,320 km in length (Bartozek et al. 2016). This river rises in the Serra do Mar and flows through a geological fault in the three plateaus in Paraná. The river flows through three regions: the upper Iguaçu on the first plateau; the middle Iguaçu on the second plateau, and the Lower Iguaçu on third plateau (Maack 1981). Before joining the Paraná River near the city of Foz do Iguaçu, the river passes over the Iguaçu Falls (Maack 1981). The falls are within INP and are the most important feature of the park. The Iguaçu Falls form a natural barrier in the Iguaçu river basin that has isolated the ichthyofauna of the Iguaçu basin from Paraná river for millions of years (Agostinho et al. 2003). This isolation has resulted in speciation and high endemism of the fish fauna in the Iguaçu basin (Garavello et al. 1997; Agostinho et al. 1999), which is estimated at 70% (Baumgartner et al. 2012).

**Figure 1.**
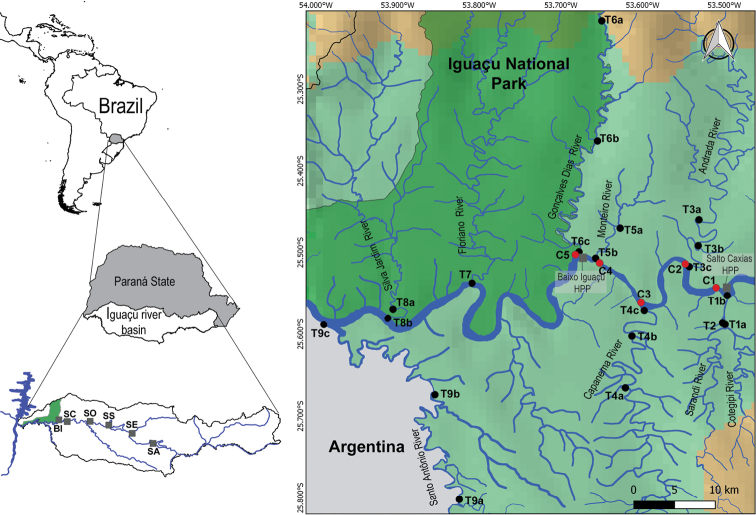
Study area: Lower Iguaçu river basin highlighting the existing hydroelectric dams (SA: Foz do Areia; SE: Segredo; SS Salto Santiago; SO: Salto Osório; SC: Salto Caxias; and BI: Baixo Iguaçu) and the Iguaçu National Park (left). Sampling sites are located in tributaries (black dots) and the main channel (red dots) of the Iguaçu River (right). Sampling sites in tributaries were indicated considering their upstream (a), intermediate (b), and downstream location (c).

The study area comprises the Lower Iguaçu River, including its tributaries and the main channel, extending from the Salto Caxias dam downstream to the mouth of the Santo Antônio mouth, which is in INP (Fig. 1) at the Brazil–Argentina boundary. In this region, 25 sites were sampled: five in the main channel and 20 in tributaries. The sampled tributaries were: Cotejipe, Sarandi, Andrada, Capanema, Monteiro (outside INP), Santo Antônio, Gonçalves Dias (boundary of INP), Floriano, and Silva Jardim rivers (within INP; Table 1).

**Table 1.** Characteristics of the sampled sites in the Lower Iguaçu river basin, Brazil. INP = Iguacu National Park; T = tributary; C = main channel.

Sites	Sub sites	Latitude and longitude	Altitude (m)	river width (m)	Description
T1	a	25°35'17.04"S, 53°29'56.58"W	257	39	Cotejipe River, tributary of Iguaçu. Located just downstream of Salto Caxias HPP.
	b	25°33'9.54"S, 53°29'46.92"W	270		
T2		25°35'10.74"S, 53°30'7.44"W	278	12	Sarandi River, tributary of Cotejipe River.
C1		25°32'30.18"S, 53°30'37.98"W	268	348	Iguaçu River, just downstream of the Salto Caxias.
T3	a	25°27'36.18"S, 53°31'51.69"W	291	24	Andrada River, tributary of Iguaçu River.
	b	25°29'29.70"S, 53°31'55.08"W	263	37	
	c	25°31'2.28"S, 53°32'34.44"W	309	62	
C2		25°30'48.00"S, 53°32'40.62"W	246	652	Iguaçu River.
T4	a	25°39'54.84"S, 53°37'15.66"W	268	25	Capanema River, tributary of Iguaçu River.
	b	25°36'8.40"S, 53°36'46.98"W	275	38	
	c	25°34'16.26"S, 53°35'52.68"W	256	72	
C3		25°33'49.14"S, 53°36'16.92"W	284	592	Iguaçu River.
C4		25°30'42.58"S, 53°39'5.76"W	262	287	Iguaçu River, just upstream of Baixo Iguaçu HPP (current reservoir).
T5	a	25°28'12.96"S, 53°37'39.00"W	269	9	Monteiro River, tributary of Iguaçu River.
	b	25°30'25.38"S, 53°39'27.24"W	279	17	
T6	a	25°12'58.98"S, 53°39'0.06"W	460	17	Gonçalves Dias River, tributary of Iguaçu River. Located at the limit of the INP (right margin). Its mouth with Iguaçu is approximately 500 meters from the Baixo Iguaçu HPP.
	b	25°21'48.12"S, 53°39'18.00"W	293	36	
	c	25°29'57.06"S, 53°40'40.50"W	241	38	
C5		25°29'57.54"S, 53°40'53.52"W	249	747	Iguaçu River, just downstream of the Baixo Iguaçu HPP reservoir, right bank in the INP.
T7		25°32'14.82"S, 53°48'31.98"W	225	39	Floriano River, a tributary of Iguaçu River. Fully inserted in the INP.
T8	a	25°34'11.09"S, 53°54'20.36"W	250	31	Silva Jardim River, a tributary of Iguaçu River. Fully inserted in the INP.
	b	25°34'51.24"S, 53°54'43.68"W	229	20	
T9	a	25°48'6.28"S, 53°49'28.35"W	265	40	Santo Antônio River, a tributary of Iguaçu River. It is the border between Brazil and Argentina.
	b	25°40'25.80"S, 53°51'15.90"W	233	15	
	c	25°35'17.16"S, 53°59'25.20"W	215	57	

The Baixo Iguaçu HPP (25°30'S, 53°40'W), the last hydroelectric power plant on the Iguaçu River downstream from Salto Caxias HPP, is approximately 500 meters from the mouth of the Gonçalves Dias River, at the INP boundary. On its right bank is the municipality of Capanema, and on its left bank is the municipality of Capitão Leonidas Marques (Paraná, Brazil).

### Data collection

Fish samples were collected (Fig. 1) using several types of fishing gear: gill nets (mesh sizes 2.5–14.0 cm), trammel nets (6.0, 7.0, and 8.0 cm), and longlines. The gear was installed and remained in position for 24 h and inspected every 6 h. Samplings were taken monthly in two periods: during the fish faunal survey from January to December 2010, and during four years of monitoring from September 2013 to March 2015, August 2015 to March 2016, and August to December 2016 (44 samplings in total).

After capture, the fish were euthanized with 250 mg/L benzocaine, fixed in 10% formaldehyde, and preserved in 70% ethanol. Fish were collected under license from the Instituto Ambiental do Paraná (IAP) (licenses no. 37788 and 43394) and Instituto Chico Mendes de Conservação da Biodiversidade (ICMBio) (no. 003/2014 and 63/2016-DIBIO/ICMBio). The protocols of the Ethics Committee on Animal Use (CEUA, no. 62/09) of the Universidade Estadual Oeste do Paraná were followed.

The specimens identified according to Baumgartner et al. (2012), Garavello et al. (2012), Garavello and Sampaio (2010), and Graça and Pavanelli (2007), and total and standard lengths (in cm) were measured. Taxonomic classification and species names mainly follow Fricke et al. (2020). Voucher specimens were deposited at the fish collection of the Museum of Zoology (MZUEL) at the Universidade Estadual de Londrina.

The species were classified according to body size, origin, and conservation status. Using standard length (measured and reported in the literature), the species were classified as small (S = <20 cm), medium (M = 20–40 cm), and large (L = >40 cm) following Baumgartner et al. (2012). For their biogeographic origins, the species were categorized following Langeani et al. (2007): autochthonous (native species that occur in other river basins), allochthonous (introduced species belonging to the Neotropical region), endemic (species restricted to the Iguaçu river basin above the Iguaçu Falls), exotic (introduced species from other continents), and hybrids (crosses of species). The origins of each species were determined according to Reis et al. (2003), Langeani et al. (2007), Baumgartner et al. (2012), and Casciotta et al. (2016).

The conservation status of species was based on the Red Book of Endangered Brazilian Fauna (ICMBio 2018), which classifies the risk of extinction of species following the International Union for Conservation of Nature (IUCN) criteria; the categories are: Extinct in the wild (EW), Critically endangered (CR), Endangered (EN), Vulnerable (VU), Near Threatened (NT), Data Deficient (DD), and Least Concern (LC).

### Data analysis

The generalized linear mixed models (GLMMs) were used to verify differences in the relative numerical abundance of species according to their origins (allochthonous, autochthonous, endemic, exotic, and hybrid) among sites. GLMMs were constructed using Gaussian family distribution, including sites as response variables (fixed factor), and the time (sampling years) as random factor. GLMMs were ran using the following packages: “nlme” (Pinheiro et al. 2021), “lme4” (Bates et al. 2015), “lmerTest” (Kuznetsova et al. 2017), “stats” (R Core Team 2021), and “car” (Fox and Weisberg 2019). When the result was significant for the categorical factor (sites), we performed a post-hoc test using the *difflsmeans* function.

To determine fish species indicative for each site category (main channel: C1–C5; tributaries outside of INP: T1-T5 and T9, and tributaries inside or in the border of INP: T6–T8), the indicator value analysis (IndVal; Dufrêne and Legendre 1997) was applied based on the relative numerical abundance of fish species using the *multipatt* function, with 999 permutations, in the “indicspecies” package v. 1.7.8 (Cáceres and Legendre 2009). Indicator values reflect specificity (the probability of a taxon occurring in a group) and fidelity (the relative abundance of the taxon in that group). IndVal produces an indicator species value (ISV) that ranges from 0 (absent) to 1 (present in all samples of a particular group). Species considered the “best” indicators of a group are those with scores closest to 1, meaning they are found within their group only and do not occur anywhere else. All statistical analyses were performed in R version 3.5.2 (R Core Team 2021), considering the confidence interval of *p* < 0.05.

## Results

**Figure 2.**
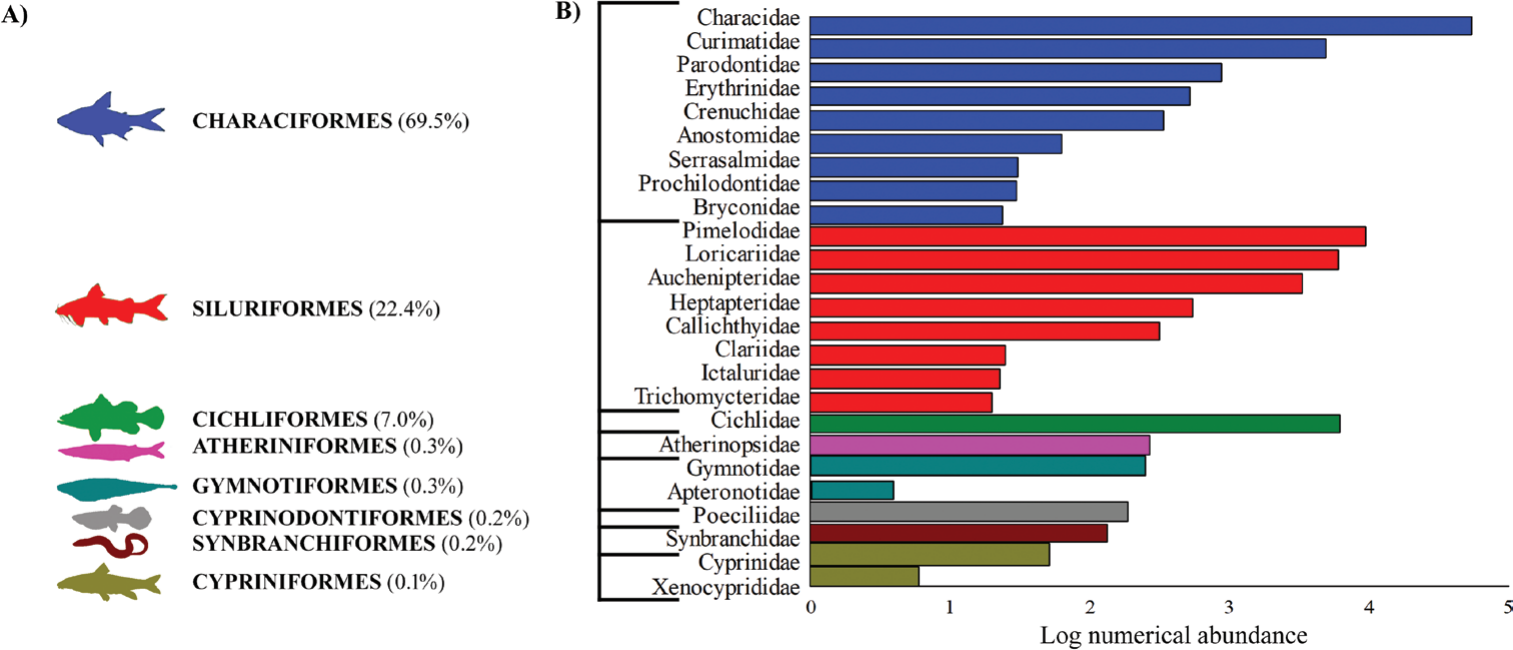
Relative numerical abundance of fish orders (**A**) and fish families (**B**) recorded between 2010 and 2016 in the Lower Iguaçu river basin, Brazil.

A total of 87,702 specimens were recorded, comprising 76 species, 25 families, 53 genera, and eight orders (Fig. 2; Table 2). The richest orders were Siluriformes and Characiformes, with 28 and 27 species, respectively (Table 2). Together these two orders represent approximately 92% of all species collected (Fig. 2a). Characidae (13 species), Cichlidae (11 species), and Loricariidae (nine species) were the families with the greatest richness (Table 2). However, Characidae, Cichlidae, and Pimelodidae are the most abundant families and comprising approximately 80% (Fig. 2b). Seven species were identified to only the genus level: *Apteronotus* sp., *Characidium* sp., *Crenicichla* sp., *Heptapterus* sp., *Hoplias* sp. 1, *Hoplias* sp. 2, and *Pariolius* sp.

**Table 2.** Fish species recorded and their respective occurrence at the sampling sites in the Lower Iguaçu River basin, Brazil. %N: abundance in numerical percentage; SL: standard lengths (minimum-maximum; cm); Size: the reported size that the species can reach: Small (S)= fish less than 20 cm; Medium (M)= 20-40 cm; and Large (L)= more than 40 cm; Origin refers to species classified in Autochthonous (AU), Endemic (END), Allochthonous (AL), Exotic (EX), and Hybrid (HY) to the Lower Iguaçu River; Threat level= Brazilian Red List of Threatened Species: Extinct in the wild (EW), Critically endangered (CR), Endangered (EN), Vulnerable (VU), Near Threatened (NT), Data Deficient (DD), and Least Concern (LC) (ICMBio 2018); Voucher specimens: individuals deposited in the Zoology Museum at the Universidade Estadual de Londrina (MZUEL). T = tributary; C = main channel.

Taxonomic position/Species	%	SL (cm) / Size	Origin/	Sampling sites														Voucher specimens
	N		Threat level	T1	T2	C1	T3	C2	T4	C3	C4	T5	T6	C5	T7	T8	T9	
** CYPRINIFORMES **																		
** Cyprinidae **																		
*Cyprinus carpio* Linnaeus, 1758	0.06	16.0/74.0/L	EX	x			x		x		x	x	x			x	x	MZUEL13303
**Xenocyprididae**																		
*Ctenopharyngodon idella* (Valenciennes, 1844)	0.01	23.0/48.8/L	EX	x			x										x	
*Hypophthalmichthys nobilis* (Richardson, 1845)	*	26.0/M	EX														x	MZUEL15861
** CHARACIFORMES **																		
** Parodontidae **																		
*Apareiodon vittatus* Garavello, 1977	1.00	1.4/15.5/S	END/LC	x	x	x	x	x	x	x	x	x	x	x	x	x	x	MZUEL17679
** Curimatidae **																		
Cyphocharax cf. santacatarinae (Fernández-Yépez, 1948)	2.67	1.3/22.7/M	AU/LC	x	x	x	x	x	x	x	x	x	x	x	x	x	x	MZUEL16272
*Steindachnerina brevipinna* (Eigenmann & Eigenmann, 1889)	2.87	2.0/22.0/M	AU/LC	x	x	x	x	x	x	x	x	x	x	x	x	x	x	MZUEL17613
** Prochilodontidae **																		
*Prochilodus lineatus* (Valenciennes, 1837)	0.03	19.6/36.0/M**	AL/LC	x		x	x		x	x		x	x		x	x	x	MZUEL13315
** Anostomidae **																		
*Megaleporinus macrocephalus* Garavello & Britski, 1988	0.03	15.3/39.6/M	AL/LC	x			x	x	x	x	x	x			x	x	x	MZUEL15870
*Megaleporinus piavussu*Britski, Birindelli & Garavello, 2012	0.02	16.4/41.2/L	AL/LC	x			x		x			x	x			x		MZUEL17944
*Megaleporinus obtusidens* (Valenciennes, 1837)	0.02	16.0/43.0/L	AL/LC	x		x	x		x	x	x		x		x	x	x	MZUEL15836
*Schizodon borellii* (Boulenger, 1900)	*	29.5/35.0/M	AL/LC			x										x		MZUEL17941
** Crenuchidae **																		
*Characidium* sp.	0.38	1.7/9.9/S	END	x	x	x	x	x	x	x	x	x	x	x	x	x	x	MZUEL17568
** Serrasalmidae **																		
*Piaractus mesopotamicus* (Holmberg, 1887)	0.04	10.5/68.0/L	AL/NT	x		x	x	x	x		x	x				x		MZUEL17986
** Characidae **																		
*Astyanax dissimilis* Garavello & Sampaio, 2011	3.14	2.0/14.4/S	END/LC	x	x	x	x	x	x	x	x	x	x	x	x	x	x	MZUEL16339
*Astyanax lacustris* Lütken, 1875	6.69	1.0/16.4/S	AL/LC	x	x	x	x	x	x	x	x	x	x	x	x	x	x	MZUEL16359
*Astyanax minor* Garavello & Sampaio, 2010	5.50	2.2/28.7/M	END/LC	x	x	x	x	x	x	x	x	x	x	x	x	x	x	MZUEL16346
*Astyanax serratus* Garavello & Sampaio, 2011	*	9.7/13.0/S	END/LC			x											x	MZUEL15827
*Bryconamericus ikaa* Casciotta, Almirón & Azpelicueta, 2004	10.83	0.7/8.3/S	END/LC	x	x	x	x	x	x	x	x	x	x	x	x	x	x	MZUEL17521
*Bryconamericus pyahu* Azpelicueta, Casciotta & Almirón, 2003	0.08	2.3/5.8/S	END/LC	x	x		x	x	x	x		x	x	x	x	x	x	MZUEL15830
*Charax stenopterus* Fowler, 1932	0.01	6.9/9.6/S	AL/LC													x	x	MZUEL13309
Diapoma aff. alburnus (Hensel, 1870)	2.40	1.1/30.0/M	AU/LC	x	x	x	x	x	x	x	x	x	x	x	x	x	x	MZUEL13243
*Hyphessobrycon boulengeri* Ellis, 1911	0.01	2.7/4.3/S	AU	x	x				x			x						MZUEL17979
*Oligosarcus longirostris* Menezes & Géry, 1983	4.46	2.2/36.4/M	END/LC	x	x	x	x	x	x	x	x	x	x	x	x	x	x	MZUEL17522
*Psalidodon bifasciatus* (Garavello & Sampaio, 2010)	20.71	2.0/38.9/M	AU/LC	x	x	x	x	x	x	x	x	x	x	x	x	x	x	MZUEL16267
*Psalidodon gymnodontus* (Eignmann, 1911)	7.68	2.0/16.3/S	END/LC	x	x	x	x	x	x	x	x	x	x	x	x	x	x	MZUEL16353
*Psalidodon gymnogenys* Eignmann, 1911	0.10	6.0/14.5/S	END/EN	x		x	x	x	x	x		x	x		x	x	x	MZUEL20821
** Bryconidae **																		
*Brycon hilarii* (Valenciennes, 1850)	0.01	18.0/30.6/M	AL/LC	x			x			x	x		x			x	x	MZUEL15855
*Salminus brasiliensis* (Cuvier, 1816)	0.02	18.0/41.0/L	AL/LC	x			x		x	x			x	x				MZUEL13302
** Erythrinidae **																		
*Hoplias* sp. 1	0.30	5.5/48.2/L	AU	x	x	x	x	x	x	x	x	x	x	x	x	x	x	MZUEL13264
*Hoplias* sp. 2	0.30	5.5/52.0/L	AU	x	x	x	x	x	x	x	x	x	x	x	x	x	x	MZUEL17662
** SILURIFORMES **																		
** Trichomycteridae **																		
*Cambeva davisi* (Haseman, 1911)	0.01	3.8/13.4/S	AU/LC									x	x					MZUEL15841
*Cambeva stawiarski* (Miranda Ribeiro, 1968)	0.01	3.5/13.0/S	END/LC									x	x					MZUEL17950
** Callichthyidae **																		
*Corydoras carlae* Nijssen & Isbrücker, 1983	*	5.5/6.0/S	END/LC						x								x	MZUEL17500
*Corydoras ehrhardti* Steindachner, 1910	0.09	1.7/4.5/S	AU/LC	x	x	x	x	x	x					x			x	MZUEL17475
*Corydoras longipinnis* (Jenyns, 1842)	0.27	1.5/14.6/S	END/LC	x	x	x	x	x	x		x	x	x	x		x	x	MZUEL17681
** Loricariidae **																		
*Ancistrus agostinhoi* Bifi, Pavanelli & Zawadzki, 2009	*	4.8/12.0/S	END/LC						x			x					x	MZUEL15856
*Ancistrus mullerae* Bifi, Pavanelli & Zawadzki, 2009	1.22	1.5/16.1/S	END/LC	x	x	x	x		x	x		x	x	x	x	x	x	MZUEL15862
*Hisonotus yasi* (Almirón, Azpelicueta & Casciotta, 2004)	0.11	1.2/19.0/S	END	x	x		x	x	x	x	x	x	x	x	x		x	
*Hypostomus albopunctatus* (Regan, 1908)	0.03	11.0/35.5/M	AU/LC	x		x	x	x	x				x				x	MZUEL15849
*Hypostomus commersoni* Valenciennes, 1836	0.17	3.3/43.5/L	AU/LC	x		x	x	x	x	x	x	x	x	x		x	x	MZUEL15887
*Hypostomus derbyi* (Haseman, 1911)	0.53	13.8/40.5/L	AU/LC	x	x	x	x	x	x	x	x	x	x	x	x	x	x	MZUEL17495
*Hypostomus myersi* (Gosline, 1947)	3.29	13.4/37.5/M	AU/LC	x	x	x	x	x	x	x	x	x	x	x	x	x	x	MZUEL16348
Loricariichthys cf. rostratus Reis & Pereire, 2000	1.44	5.0/28.5/M	AU/LC	x		x	x	x	x	x	x	x	x	x	x	x	x	MZUEL17604
Pareiorhaphis cf. parmula Pereira, 2005	*	2.5/2.5/S	END/LC										x					
** Heptapteridae **																		
*Heptapterus* sp.	*	11.0/16.0/S	END		x													MZUEL15845
*Imparfinis hollandi* Haseman, 1911	0.02	3.7/25.8/M	END	x	x		x	x				x	x				x	MZUEL17985
*Pariolius* sp.	0.01	8.5/18.5/S	END		x							x						
*Rhamdia branneri* Haseman, 1911	0.19	6.3/39.0/M	END/LC	x	x	x	x	x	x	x		x	x	x	x	x	x	MZUEL13276
*Rhamdia voulezi* Haseman, 1911	0.41	5.0/36.8/M	END/LC	x	x	x	x	x	x	x	x	x	x	x	x	x	x	MZUEL15871
** Ictaluridae **																		
*Ictalurus punctatus* (Rafinesque, 1818)	0.03	11.0/73.8/L	EX	x			x	x	x			x				x	x	MZUEL13246
** Auchenipteridae **																		
*Glanidium ribeiroi* Haseman, 1911	3.75	5.1/29.0/M	END/LC	x	x	x	x	x	x	x	x	x	x	x	x	x	x	MZUEL16268
*Tatia jaracatia* Pavanelli & Bifi 2009	0.10	3.9/7.4/S	END/LC	x	x	x	x	x	x	x	x	x	x	x		x	x	MZUEL16278
** Clariidae **																		
*Clarias gariepinus* (Bourchell, 1822)	0.03	19.8/85.0/L	EX	x			x	x	x	x	x					x	x	MZUEL15858
** Pimelodidae **																		
*Leiarius marmoratus* (Gill, 1870)	*	35.5/35.5/M	AL/LC						x									MZUEL15874
*Pimelodus britskii* Garavello&Shibatta, 2007	10.12	1.0/40.2/L	END/LC	x	x	x	x	x	x	x	x	x	x	x	x	x	x	MZUEL17494
*Pimelodus ortmanni* Haseman, 1911	0.79	9.0/32.0/M	END/LC	x	x	x	x	x	x	x	x	x	x	x	x	x	x	MZUEL16275
*Pseudoplatystoma corruscans* (Spix& Agassiz, 1829)	*	42.5/58.0/L	AL/NT													x	x	MZUEL20820
*Steindachneridion melanodermatum* Garavello, 2005	0.01	17.4/72.5/L	END/EN			x			x				x				x	MZUEL17620
** GYMNOTIFORMES **																		
** Gymnotidae **																		
*Gymnotus inaequilabiatus* (Valenciennes, 1839)	0.04	8.0/21.4/M	AL/LC	x			x		x			x	x	x			x	MZUEL16279
*Gymnotus sylvius* Albert & Fernandes-Matioli, 1999	0.25	2.5/34.0/M	AL/LC	x	x	x	x	x	x	x	x	x	x	x	x	x	x	MZUEL13300
** Apteronotidae **																		
*Apteronotus* sp.	*	26.7/27.5/M	AU				x										x	MZUEL13271
** ATHERINIFORMES **																		
** Atherinopsidae **																		
*Odontesthes bonariensis* (Valenciennes, 1835)	0.31	4.8/34.5/M	AL/DD	x		x	x	x	x	x	x	x				x	x	MZUEL13290
** CYPRINODONTIFORMES **																		
** Poeciliidae **																		
*Phalloceros harpagos* Lucinda, 2008	0.21	1.0/4.1/S	AU/LC	x	x		x	x	x		x	x	x		x	x	x	MZUEL17981
*Poecilia reticulata* Peters, 1859	*	1.4/1.7/S	AL									x						MZUEL15839
** SYNBRANCHIFORMES **																		
** Synbranchidae **																		
*Synbranchus marmoratus* Bloch, 1795	0.15	6.8/41.0/L	AL/LC	x	x	x	x		x		x	x	x	x	x	x	x	MZUEL13245
** CICHLIFORMES **																		
** Cichlidae **																		
*Australoheros kaaygua* Casciotta, Almirón & Gómez, 2006	0.01	2.7/9.0/S	END/LC										x	x			x	MZUEL15854
*Coptodon rendalli* (Boulenger, 1897)	0.07	3.2/42.3/L	EX	x	x	x	x	x	x	x	x	x	x		x	x	x	MZUEL16254
*Crenicichla iguassuensis* Haseman, 1911	2.68	1.8/36.6/M	END/LC	x	x	x	x	x	x	x	x	x	x	x	x	x	x	MZUEL17614
*Crenicichla lepidota* Heckel, 1840	0.06	4.8/17.2/S	AU/LC	x			x	x	x				x			x	x	MZUEL15847
*Crenicichla* sp. Casciotta, Almirón & Gómez, 2006	0.97	2.0/29.1/M	AU/LC	x	x	x	x	x	x	x	x	x	x	x	x	x	x	MZUEL13301
*Crenicichla tapii* (Piálek, Dragová, Casciotta, Almirón y Rícan, 2015)	*	10.0/10.0/S	END										x					MZUEL20809
*Crenicichla tesay* Casciotta & Almirón, 2009	0.15	3.0/19.6/S	END/LC	x	x	x	x	x	x	x		x	x	x	x	x	x	MZUEL20811
*Crenicichla tuca* (Piálek, Dragová, Casciotta, Almirón y Rícan, 2015)	*	9.6/9.6/S	END												x			MZUEL20810
*Geophagus iporangensis* Haseman, 1911	2.28	1.1/41.5/L	AU/LC	x	x	x	x	x	x	x	x	x	x	x	x	x	x	MZUEL17616
*Gymnogeophagus taroba* Casciotta, Almirón, Piálek & Rican, 2017	0.73	1.3/11.1/S	END/EN	x	x	x	x	x	x	x	x	x	x	x	x	x	x	MZUEL16354
*Oreochromis niloticus* (Linnaeus, 1758)	0.09	3.3/43.0/L	EX	x	x	x	x	x	x	x		x	x	x		x	x	MZUEL13318
**Hybrid**																		
*Piaractus mesopotamicus* X *Colossoma macropomum*	*	33.5/36.9/M	HY	x			x											MZUEL15832
*Piaractus mesopotamicus* X *Piaractus brachypomus*	*	31.6/31.6/M	HY							x								
*Pseudoplatystoma corruscans* X *Pseudoplatystoma fasciatum*	*	28.0/46.0/L	HY												x	x		MZUEL15877

* Relative numerical abundance (%) smaller than 0.01 ** Species less than 40 cm in length, but considered large in the literature (Baumgartner et al. 2012).

Species richness was greater (76 species) at sites in the tributaries than in the main channel (58 species). The tributaries with the highest species richness were T9 (62 species) and T4 (58 species). Species richness was less in T7 (39 species). Eighteen species were caught only in tributaries. The following species had a restricted occurrence: *Heptapterus* sp. in T2, *Leiarius marmoratus* in T4, *Poecilia reticulata* in T5, Pareiorhaphis cf. parmula and *Crenicichla tapii* in T6, *Crenicichla tuca* in T7, and *Hypophthalmichthys nobilis* in T9. In the main channel, the greatest species richness was at C2 (46 species) and the lowest at C4 and C5 (40 species each). The hybrid *Piaractus mesopotamicus* × *P. brachypomus* had restricted capture in the main channel (C3). The most frequent species at all sampling sites (main channel and tributaries) were *Psalidodon bifasciatus* (21%), *Bryconamericus ikaa* (11%), and *Pimelodus britskii* (10%).

The fish fauna was characterized chiefly by small and medium-sized species (74% of total numerical abundance; Table 2), represented mainly by *Psalidodon bifasciatus* (24%), *P. gymnodontus* (13%), and *B. ikaa* (9%). Nineteen large species were shared between the main channel and tributaries, with *P. britskii* (71%) being the most frequent and *Steindachneridion melanodermatum* the rarest (Table 2). Some large species were recorded only in the tributaries: *Ctenopharyngodon idella*, *Megaleporinus piavussu*, and *Pseudoplatystoma corruscans*.

On the biogeographic origin of the species in terms of richness, 42% are endemics, 24% autochthonous, 21% allochthonous, 9% exotic, and 4% hybrids. In terms of abundance, endemic and autochthonous species represented 92% of the total abundance (54% and 38%, respectively). In general, the most frequent endemic species were *B. ikaa* (10.83%), *P. britskii* (10.12%), and *P. gymnodontus* (7.68%). *Psalidodon bifasciatus* (20.71%) was most frequent autochthonous species, *Astyanax lacustris* (6.69%) the most frequent allochthonous species, *Oreochromis niloticus* (0.09%) and *Coptodon rendalli* (0.07%) the most frequent exotic species, and *Pseudoplatystoma corruscans* × *P. fasciatum* (<0.001%) was the most frequent hybrid (Table 2).

**Figure 3.**
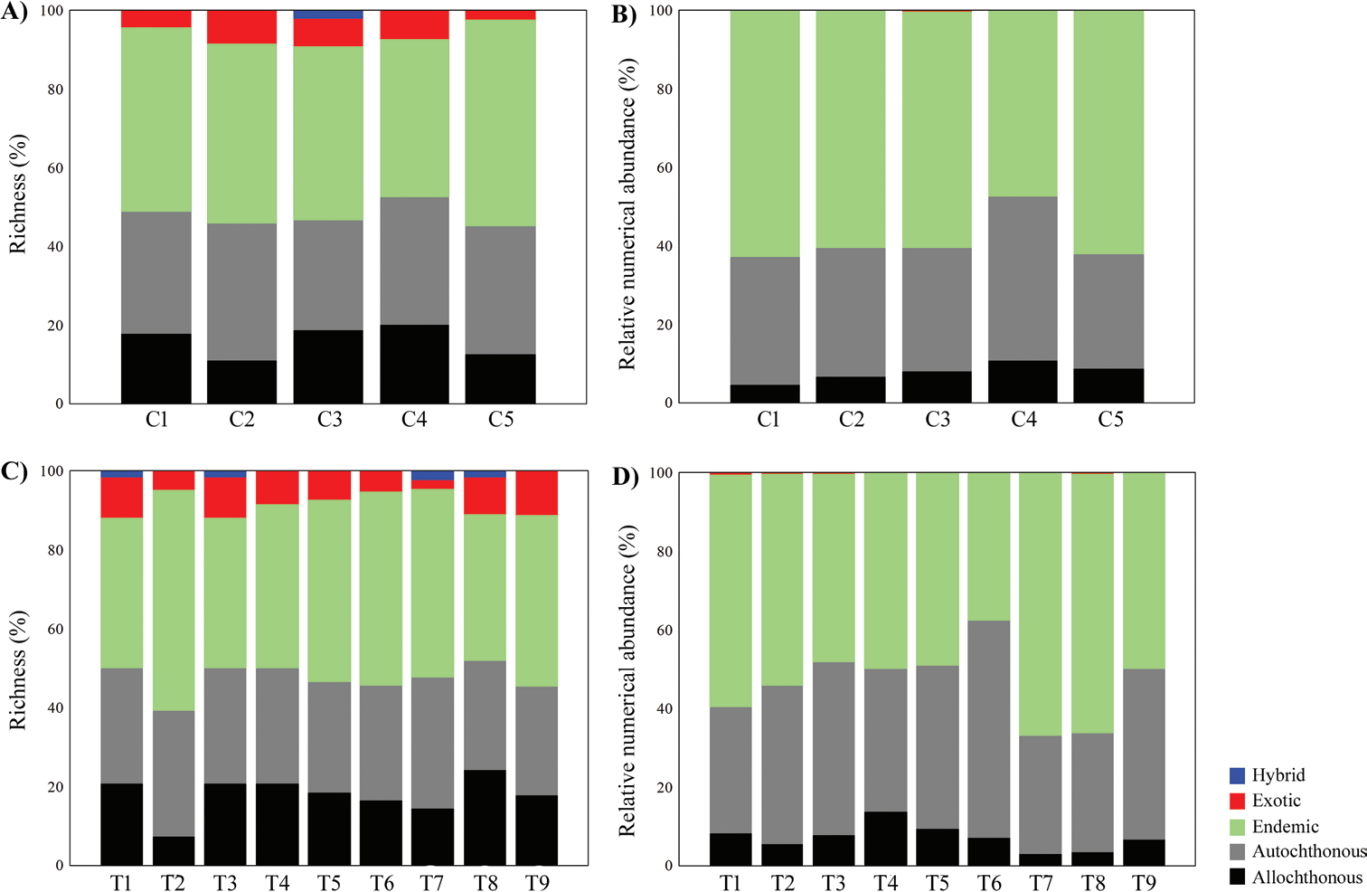
Richness (%, **A–C**) and relative numerical abundance (**B–D**) of fish species according to the origin (AL: allochthonous; AU: autochthonous; END: endemic; EX: exotic; HY: hybrid) recorded between 2010 and 2016 in the tributaries (**C, D**) and main channel (**A, B**) Lower Iguaçu river basin, Brazil.

The results of the GLMMs indicated that the relative numerical abundance of allochthonous (*F* = 2.54; *p* = 0.007), autochthonous (*F* = 3.80; *p* = 0.0001), and endemic (*F* = 4.30; *p* < 0.0001) species differed among sites (Table 3; Fig. 3). For exotic species and hybrids, there were no significant relationships with sites (*F* = 1.32; *p* = 0.23; *F* = 0.97; *p* = 0.49, respectively). The main channel (C1 and C4) and tributaries (T2, T3, T4, T6, and T9) were the sites related with higher abundance of endemic species. In addition, C1, C4 and tributaries (T3, T6, and T9) also related to a great abundance of autochthonous species, and the main channel (C1 and C4) and tributaries outside of INP (T4 and T5) were most abundant in allochthonous species. Despite non-significant results, exotic and hybrid species were also richer and highly abundant in the tributaries, especially in those areas outside of INP, and in areas with intense urban and agricultural activities (Fig. 3; Table 2). The indicator species analysis (Table 4) showed that, among the 76 species considered, only a few species were significantly related with biogeographic origin: *O. bonariensis* (allochthonous) was an indicator species of the main channel, *P. harpagos* (autochthonous) and *I. punctatus* (exotic) were indicator species of tributaries located outside of INP, and *A. mullerae* (endemic) and *R. branneri* (endemic) were indicator species of tributaries inside INP.

**Table 3.** Effects of the sampling sites on the relative numerical abundance of autochthonous, allochthonous, and endemic species evaluated in the generalized linear mixed models (GLMMs).

Sites	Endemic					Autochthonous					Allochthonous					
	Estimate	Std. Error	df	t value	Pr(>|t|)	Estimate	Std. Error	df	t value	Pr(>|t|)	Estimate	Std.	Error	df	t value	Pr(>|t|)
C1	63.97	4.20	56.08	15.24	**< 0.0001**	30.64	3.77	63.08	8.12	**< 0.0001**	5.20	5.20	1.68	56.44	3.09	**0.003**
C2	-4.35	5.90	59.28	-0.74	0.464	2.62	5.51	59.35	0.48	0.636	1.97	1.97	2.36	59.50	0.83	0.409
C3	-1.52	5.90	59.28	-0.26	0.797	-1.66	5.51	59.35	-0.30	0.764	3.27	3.27	2.36	59.50	1.38	0.172
C4	-17.99	5.61	58.87	-3.21	**0.002**	12.01	5.24	58.69	2.29	**0.026**	6.12	6.12	2.25	59.12	2.72	**0.008**
C5	-6.30	5.61	58.87	-1.12	0.266	3.72	5.24	58.69	0.71	0.481	2.69	2.69	2.25	59.12	1.20	0.237
T1	1.97	5.61	58.87	0.35	0.727	-3.91	5.24	58.69	-0.75	0.459	1.71	1.71	2.25	59.12	0.76	0.450
T2	-11.77	5.61	58.87	-2.10	**0.040**	9.11	5.24	58.69	1.74	0.088	2.70	2.70	2.25	59.12	1.20	0.235
T3	-17.63	5.90	59.28	-2.99	**0.004**	15.07	5.51	59.35	2.74	**0.008**	2.67	2.67	2.36	59.50	1.13	0.262
T4	-13.14	5.61	58.87	-2.34	**0.023**	5.34	5.24	58.69	1.02	0.313	7.80	7.80	2.25	59.12	3.47	**0.001**
T5	-11.14	5.61	58.87	-1.99	0.052	6.66	5.24	58.69	1.27	0.209	4.57	4.57	2.25	59.12	2.03	**0.047**
T6	-22.91	5.90	59.28	-3.89	**< 0.0001**	20.74	5.51	59.35	3.77	**< 0.0001**	2.10	2.10	2.36	59.50	0.89	0.378
T7	0.57	5.90	59.28	0.10	0.923	0.62	5.51	59.35	0.11	0.911	-1.16	-1.16	2.36	59.50	-0.49	0.624
T8	3.30	5.90	59.28	0.56	0.578	-1.54	5.51	59.35	-0.28	0.780	-1.97	-1.97	2.36	59.50	-0.84	0.407
T9	-17.55	5.61	58.87	-3.13	**0.003**	16.71	5.24	58.69	3.19	**0.002**	0.89	0.89	2.25	59.12	0.40	0.693

**Table 4.** Species indicators defined by IndVal analysis, performed for main channel and tributaries outside and inside Iguaçu National Park – INP.

Species indicator	stat	*p*
	**Main channel**	
*O. bonariensis*	0.70	0.001
	**Tributaries outside of INP**	
*P. harpagos*	0.74	0.001
*I. punctatus*	0.49	0.024
	**Tributaries inside of INP**	
*A. mullerae*	0.95	0.001
*R. branneri*	0.74	0.004

Three Endangered (EN) species were sampled in low abundance (<1%) (Table 2): *Psalidodon gymnogenys*, captured at most sites in the tributaries and the main channel but especially at C1 (0.38%); *Steindachneridion melanodermatum*, captured at T4, T6, and T9 in the tributaries but principally in the main channel at C1 (0.07%), and *Gymnogeophagus taroba*, captured widely in the study but especially at T9.

## Discussion

This study is the first ichthyofaunistic survey carried out on a dam-free stretch of the Iguaçu River and its tributaries between the Salto Caxias Dam and the Iguaçu Falls. The number of identified species accounted for 72% of the number of species observed in a previous study for the Lower Iguaçu basin (Baumgartner et al. 2012), of which seven species had not been recorded (*Schizodon borellii*, *Charax stenopterus*, *Leiarius marmoratus*, *Poecilia reticulata*, *Crenicichla lepidota*, *C. tapii*, and *C. tuca*). Other species identified only to genus level still have unresolved taxonomy (*Apteronotus* sp., *Characidium* sp., *Heptapterus* sp., *Hoplias* sp. 1, *Hoplias* sp. 2, and *Pariolius* sp.). These results are important, as the stretch of river studied by Baumgartner et al. (2012) was over 250 km long and included five reservoirs upstream of our study area. The high species richness we found may be due, in part, to the unprecedent collections within a conservation area, the INP.

The richness and abundance of Siluriformes and Characiformes species were higher than those of other orders, both in the Iguaçu River and in its tributaries. Similarly, the same pattern was pointed out by previous studies along the Lower Iguaçu river basin: in reservoirs (Baumgartner et al. 2006), in rivers (Bifi et al. 2006), and in streams (Sereia et al. 2017; Delariva et al. 2018). This pattern in the Iguaçu river basin demonstrates a trend in many Neotropical watersheds, as observed by Lowe-McConnell (1999).

Small water bodies are as refuges for small species and provide a greater diversity of food resources from riparian vegetation and a larger diversity of microhabitats (Castro and Polaz 2020). Our study finds a more remarkable small-bodied species richness in tributaries than in the main channel. Additionally, the results of GLMM also showed the tributaries importance for conserving endemic species, both outside of (T2, T3, T4, T9) and inside INP (T6). The autochthonous Pareiorhaphis cf. parmula and *C. tapii* were recorded only in tributaries within INP (T6), which suggests the park’s role in the conservation of the fish fauna. Other species also had restricted capture in tributaries, but outside INP: the autochthonous *Heptapterus* sp. (T2), the allochthonous *P. reticulata* (T5), and *L. marmoratus* (T4), and the exotic *Hypophthalmichthys nobilis* (T9), indicating that tributaries without the protection afforded by being outside of the INP are more susceptible to anthropic threats.

Other small species, mainly belonging to the genera *Astyanax*, *Psalidodon*, and *Crenicichla*, occurred at all sampling sites. These species are generalists with high trophic plasticity, favoring their wide distribution within the basin and in varied habitats (Pini et al. 2019; Delariva and Neves 2020; Kuhn et al. 2020). Some *Astyanax* species were described in the last decades (Alcaraz et al. 2009; Garavello and Sampaio 2010), but taxonomic relationships and the identity of some of these remains uncertain (Rossini et al. 2016), caused by phenotypic plasticity (Pavanelli and Oliveira 2009), which will require full taxonomic review.

The introduction of species is among the leading causes of species extinction in worldwide (Matthews 1998), and this problem has already been highlighted in the Lower Iguaçu river basin. The transfer of these species to the Iguaçu basin has multiple reasons but may be a result of commercial and sport fishing (using live bait), aquaculture, fish stocking, and aquarium fish release (Garavello et al. 1997; Daga et al. 2016; Larentis et al. 2019). The exotic *P. reticulata* was recorded only in the Monteiro River (T5), whose basin is highly impacted by the urbanization of the city Capitão Leonidas Marques near the sampling site. Allochthonous species were also recorded elsewhere in the Iguaçu river basin, such as in the Segredo reservoir (Garavello et al. 1997) and the Salto Osório reservoir (Baumgartner et al. 2006), where the migratory *P. lineatus* was introduced. The allochthonous *Astyanax lacustris* is commonly reported for the Upper Paraná river basin, and its introduction is uncertain.

Fish farms are potential sources of invasive species (Orsi and Agostinho 1999; Daga et al. 2016) and impact the basin (Agostinho et al. 1999). The capture of the allochthonous *Salminus brasiliensis* is due to escapes and releases, possibly originating from fish farms to increase sport fishing potential, as reported by residents in the region. *Salminus brasiliensis* is considered potentially invasive and can cause serious harmful effects to the ecosystem where it is introduced (Vitule et al. 2014). The exotic Tilapia species, *Oreochromis niloticus* and *Coptodon rendalli*, were probably escapes from fish farms. Tilapia culture already has an alarmingly poor record of high-risk invasions into natural environments (Frota et al. 2019). Records of introduced species were also found in Iguaçu reservoirs (Foz do Areia, Segredo, Salto Santiago, Salto Osório and Salto Caxias) where 20 species are known, with Tilapia being among the most common (Daga and Gubiani 2012). The presence of hybrids is associated with fish farming (*Piaractus mesopotamicus* × *Colossoma macropomum*, *Piaractus mesopotamicus* × *Piaractus brachypomus*, *Pseudoplatystoma corruscans* × *Pseudoplatystoma fasciatum*) (Valladão et al. 2018).

Due to their multiple uses of water, the implementation of hydroelectric projects has also been associated with facilitating the introduction and dissemination of exotic species (Agostinho et al. 1999). In addition, changes in the river’s physical and chemical characteristics promote non-measurable pressure on fish fauna, especially for species with greater sensitivity and specific ecological requirements. *Psalidodon gymnogenys*, *Steindachneridion melanodermatum*, and *Gymnogeophagus taroba*, could be most severely affected as they are already Endangered (ICMBio 2018). *Steindachneridion melanodermatum* is the largest fish in the Iguaçu River. It is an endemic and possibly migratory (Agostinho and Gomes 1997; Ludwig et al. 2005; Brehm et al. 2016), living in fast-flowing, deep waters in stretches of the Iguaçu River and tributaries where the natural flow of water is still preserved (Garavello 2005). In addition to the losses of their habitat and connectivity caused by the successive hydroelectric dams, fishing also contributes to declines in this species population (Assumpção et al. 2017). Stocks of this species have been under pressure from prohibited fishing (Assumpção et al. 2021) and are a challenge to monitor because the species occurs in two countries (Brazil and Argentina), and the fishing is most intense on weekends and holidays (UNIOESTE 2017). The extinction of *S. melanodermatum* could harm other trophic levels as it is a top-of-the-chain species. *Gymnogeophagus taroba*, a species of fast waters (Paiz et al. 2017), is widely distributed in the studied area. However, with the construction of the new hydroelectric reservoir, the species can disappear in the flooded area, and its distribution can be fragmented, which will lead to loss of genetic diversity and a population decline (Souza-Shibatta et al. 2018).

## Conclusions

The last dam-free stretch of the Lower Iguaçu River upstream of the Iguaçu Falls exhibits a rich endemic fish fauna, rare endangered species restricted to this region, and new species for science. This diversity is threatened with extinction by biotic and abiotic factors. Exotic species have occurred in low abundance, but their presence in most sampling sites and the Iguaçu National Park is worrisome, requiring actions to mitigate its harmful effects and to avoid new introductions. The presence of hybrids of allochthonous species escaped from fish farms requires strict supervision of these commercial operations. Another source of threats is the construction of the Baixo Iguaçu HPP, which will promote hydrological changes in the main channel and severe damage to many fish species. Thus, tributaries will play an essential role in maintaining the diversity of fish in the Iguaçu river basin since many species of the Iguaçu River also frequent in the tributaries, besides the species that occur only in these environments. The protection of free-flowing tributaries has been an appeal worldwide (Grill et al. 2019; Makrakis et al. 2019), as they support endangered species populations, provide various environmental conditions, access to spawning habitat, and refugia for early life stages (Silva et al. 2019). The correct identification of species and taxonomic research are also essential, as they will help the development of strategies for the management and conservation of environments (Assumpção et al. 2021). Thus, preserving the free stretch below the Baixo Iguaçu HPP to the Iguaçu Falls is crucially necessary and the last resource to conserve endemic and endangered species. In addition, to enable the management of ichthyofauna, efforts should be concentrated on monitoring populations.
